# Quality of life in patients with psoriasis

**DOI:** 10.1192/j.eurpsy.2021.487

**Published:** 2021-08-13

**Authors:** M. Bouhamed, S. Kolsi, M. Ben Abdallah, I. Feki

**Affiliations:** Psychiatry, Hedi chaker hospital, Sfax, Tunisia

**Keywords:** quality of life, psoriasis

## Abstract

**Introduction:**

Psoriasis is a chronic inflammatory skin condition affecting diverse racial/ethnic groups throughout the world. It has a major impact on the patient’s quality of life, influencing career, social activities, family relationships, and all other aspects of life

**Objectives:**

To evalue the quality of life in patients with psoriasis

**Methods:**

Participants were outpatients of Hedi chaker University Hospital Center in sfax, Tunisia, recruited between January and July of 2017, diagnosed with psoriasis. A Demographic questionnaire and the Quality of life Questionnaire (SF-36) were administered in this study.

**Results:**

44 patients were included in this study. They had with a mean age of 45.8 ±12.1. The majority of patients were married (70.5%), unemployed (40.5%), without medical heredity (84,6%). Psoriasis was in plaque (65.9%), guttate (20.5%) and pustular(13.6.5%). Its severity assessed by BSA, was mild to moderate in 72.7% of cases and associated arthropathy was noted in 29.5% of patients. The overall average SF-36 scale scores for all patients ranged from 4 to 98 with an average of 55.97. The quality of life of patients was impaired in 45.5% of casesQuality of life was significantly more impaired in patients with associated arthropathy (p=0.004). There is no significant differences for the different dimensions of quality of life regarding the clinical form of psoriasis.

**Conclusions:**

Psoriasis certainly has an impact on patients’ quality of life.So, dermatologists should give special attention to this subgroup of persons in order to prevent future psychopathology.

